# Stabilization of a salamander moving hybrid zone

**DOI:** 10.1002/ece3.2676

**Published:** 2016-12-21

**Authors:** Michaël Visser, Maarten de Leeuw, Annie Zuiderwijk, Jan W. Arntzen

**Affiliations:** ^1^ Naturalis Biodiversity CenterLeidenThe Netherlands

**Keywords:** amphibians, enclave, habitat preferences, mosaic hybrid zone, newt, pond loss, species distribution model, *Triturus*

## Abstract

When related species meet upon postglacial range expansion, hybrid zones are frequently formed. Theory predicts that such zones may move over the landscape until equilibrium conditions are reached. One hybrid zone observed to be moving in historical times (1950–1979) is that of the pond‐breeding salamanders *Triturus cristatus* and *Triturus marmoratus* in western France. We identified the ecological correlates of the species hybrid zone as elevation, forestation, and hedgerows favoring the more terrestrial *T. marmoratus* and pond density favoring the more aquatic *T. cristatus*. The past movement of the zone of ca. 30 km over three decades has probably been driven by the drastic postwar reduction of the “bocage” hedgerow landscape, favoring *T. cristatus* over *T. marmoratus*. No further hybrid zone movement was observed from 1979 to the present. To explain the changing dynamics of the hybrid zone, we propose that it stalled, either because an equilibrium was found at an altitude of ca. 140 m a.s.l. or due to pond loss and decreased population densities. While we cannot rule out the former explanation, we found support for the latter. Under agricultural intensification, ponds in the study area are lost at an unprecedented rate of 5.5% per year, so that remaining *Triturus* populations are increasingly isolated, hampering dispersal and further hybrid zone movement.

## 1 Introduction

The interactions that determine a species' position in the ecosystem are many and include predation, competition, disease vectors, and many others. When species expand their geographical range, such as following a glacial period, they encounter new habitats and run into species previously unknown to them. If a species encounters another closely related species, the two may interbreed, possibly leading to offspring in a more or less narrow hybrid zone. At least 10% of animal species and 25% of plant species, mostly the youngest ones, are involved in hybridization and potential introgression with other species (Mallet, 2005). Zones where related species meet, mate, and hybridize are particularly frequent in areas of postglacial colonization (Hewitt, 1999; Taberlet, Fumagalli, Wust‐Saucy, & Cosson, 1998). Hybrid zones are important as a “natural laboratory” for speciation research and serve as windows on evolutionary process (Abbott et al., 2013; Harrison, 1990; Hewitt, 1988). Moving hybrid zones have an additional edge because, by moving, introgressed genes are continuously being tested in new environmental and genetic backgrounds. To exploit this asset, it is important to improve our understanding of the spatiotemporal dynamics of hybrid zones. We here investigate a unique case of a hybrid zone that moved and then stabilized in historical times. Distinguishing between stable (tension) and dynamic (moving) hybrid zones has important implications for our understanding of the role of differential introgression and selection in shaping species boundaries.

Species hybrid zones frequently show a strong ecological component. One example is the genus *Bombina* in which the lowland red‐bellied toad (*Bombina bombina*, with an Ukrainian glacial refugium) encounters the mountain dwelling yellow‐bellied toad (*Bombina variegata*, with a Balkan glacial refugium) all along the lowland–mountain transition of Central Europe (Szymura, 1993; Vörös, Mikulíček, Major, Recuero, & Arntzen, 2016). This hybrid zone could have formed along the species ecotone where it remained in stable position, but another explanation is that it formed elsewhere and then moved till the lowland–mountain transition was reached. Biogeographical evidence supports the latter scenario (Arntzen, 1978). This author documented the presence of “enclaves” of *B. variegata* surrounded by *B. bombina* and argued that in low dispersal organisms, such as toads, the only reasonable explanation for enclaves is species displacement. The same line of reasoning has been applied to myobatrachid frogs in western Australia (Littlejohn & Roberts, 1975) and to *Triturus* newts in the Iberian (Arntzen & Espregueira Themudo, 2008) and Balkan peninsulae (Wielstra & Arntzen, 2012).

**Figure 1:**
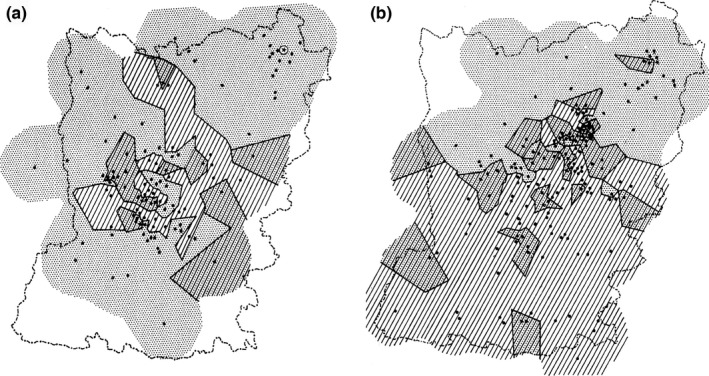
The historical distribution of the northern crested newt, *Triturus cristatus* (hatched), and the marbled newt, *Triturus marmoratus* (shaded), over the département Mayenne (a) at the first survey at ca. 1950 (data from Vallée, 1959) and (b) the second survey in 1979 (data from Schoorl & Zuiderwijk, 1981). Pond localities investigated are shown by black dots. The asterisk in the left panel has initially been attributed to a *T. cristatus* introduction, but is likely to represent a natural occurrence (see text for details). The figure is reproduced from Arntzen and Wallis (1991), with permission

Another case of a moving hybrid zone is that of the newts *Triturus cristatus* (the northern crested newt) and *Triturus marmoratus* (the marbled newt). These species engage in a habitat patchwork in central France, with adult F_1_ hybrids making up 4% of the total adult population (Arntzen, Jehle, Bardakci, Burke, & Wallis, 2009). Evidence for movement of the hybrid zone is threefold: first, through direct observation, involving the surveying of species and hybrids over a large area in ca. 1950 and in 1979. This showed the (northward) advance of *T. cristatus*, the regression of *T. marmoratus*, and the continued presence of hybrids (Schoorl & Zuiderwijk, 1981; Vallée, 1959) (Figure 1). Second, *T. marmoratus* is surrounded by *T. cristatus* in enclaves and other persisting occurrences in areas of species replacement (Arntzen & Wallis, 1991; Arntzen, 1996; see also Arntzen, Burke, & Jehle, 2010). Third, genetic variation thought to result from hybridization is significantly higher in *T. cristatus* than in *T. marmoratus* (Arntzen & Wallis, 1991). This observation is in line with the direction of hybrid zone movement.

We set out to survey the distributions of *T. cristatus*,* T. marmoratus*, and their hybrids over the French “département” Mayenne in 2014 and 2015. That is ca. 65 years after the first survey (Vallée, 1959) and 35 years after the second survey (Schoorl & Zuiderwijk, 1981) in the same area. The objective of this third survey is to document the present‐day distribution of the two newt species and make comparisons to the two previous analyses of the hybrid zone, in particular to see whether (1) *T. cristatus* continued its advance over *T. marmoratus*, (2) enclaves persisted or dissolved over time, or (3) equilibrium conditions between *T. cristatus* and *T. marmoratus* have been reached. A further aim is to identify ecological correlates of the position of the hybrid zone in situations of stasis and flux.

## 2 Materials and Methods

### 2.1 Fieldwork

Fieldwork was carried out over the département Mayenne in western France during the spring of 2014 and 2015. Ponds and other potentially suitable habitats for reproducing *Triturus* newts were located in the field with the help of digital 1:25,000 topographical maps of the “Institute National de l'Information Geographique et Forestière” (IGN). Freshly deposited *Triturus* eggs and embryos (hereafter called “eggs”) were collected from aquatic vegetation such as Plicate sweet grass (*Glyceria notata*) and water mint (*Mentha aquatic*) from the accessible corners of the pond and stored in excess 96% alcohol. Dip netting for adult newts was performed in selected ponds. Dip nets and boots were regularly cleaned with a 1% (w/v) solution of Virkon^®^ to prevent possible cross‐contamination of chytrid fungus between field sites (Dejean, Miaud, & Schmeller, 2010; Johnson, Berger, Philips, & Speare, 2003; Schmeller, Loyau, Dejean, & Miaud, 2011). To obtain an impression of the possible decline of amphibian breeding localities over time that might be interfering with the monitoring of the position of hybrid zone, we went back to pond locations documented ca. 35 and 18 years ago (Figure 1). We revisited the following: (1) the *Triturus* localities from the second survey, (2) the amphibian ponds from the second survey north of the N12 and D35 roads (AZ, unpublished data), and (3) the amphibian ponds from a survey in the Pré‐en‐Pail area in 1997 (JWA, unpublished data). We do not report on a few ponds for which we were uncertain about the exact location or where access was denied.

### 2.2 Molecular identification

DNA extraction followed the chelating resin‐based procedure of Walsh, Metzger, and Higuchi (1991). Individual eggs were placed in 1.5‐ml tubes with 0.4 ml of a 5% chelex resin solution (Chelex^®^ 100 sodium form, 50–100 mesh) and 5 μl proteinase K (ProtK, 20 mg/ml) and left overnight to lyse at 65°C. The dissolved tissue was heated at 95°C for 10 min and centrifuged for 10 min at 20,000 g. Ca. 0.3 ml of the supernatant was removed for storage at −20°C. SNP genotyping was used to identify the species of each egg. This analysis is based on the fact that the two newt species have different single nucleotide polymorphisms (SNPs) in their DNA. A common primer binds to the DNA of both species, while two allele‐specific primers attach to the DNA of either *T. cristatus* or *T. marmoratus*. These allele‐specific primers contain fluorescent labels to identify the different species. Primers were newly developed for the mitochondrial gene NADH dehydrogenase subunit 4 (ND4) as common primer 5′ GATGAAATAAGCCCGTGTGAGAT 3′ and the species‐specific primers 5′ ATTATAATTCAAACACCG 3′ for *T. cristatus* and 5′ ATTATAATTCAAACACCA 3′ for *T. marmoratus*, and for cytochrome c oxidase subunit I (COI) as common primer 5′ ATTYTAGGGGCAATTAACTTTAT 3′ and the species‐specific primers 5′ TGATGGGGGTTTTATATTAATTGATGTTGTA 3′ for *T. cristatus* and 5′ ATGGGGGTTTTATATTAATTGATGTTGTG 3′ for *T. marmoratus*. The species diagnostic nucleotides were on position 231 at the ND4 fragment (GenBank accession numbers JQ653387 and JQ653401, as in Wielstra et al., 2013) and on position 448 or 440 at the COI fragment (GenBank accession numbers KP697896, JN379828 and unpublished data JWA). The material collected in 2014 was analyzed for ND4, and the material collected in 2015 was analyzed for both COI and ND4. Because a complete match was observed between the two mtDNA markers, it was deemed unnecessary to study more than a single marker in the other material. Genotypes were called automatically by the module Kraken™ of LIMS controlling the LGC genomics SNP genotyping line, visually inspected and if necessary, manually corrected. An ambiguous signal with no call made precluded the identification of 41 eggs (3.5%).

### 2.3 No numerical correction on species counts

*Triturus cristatus* and *T. marmoratus* are dissimilar species in many respects. Relevant for data interpretation are the parameters that could affect species counts, namely (1) the length of time the adults stay in the water when reproduction is over (long in *T. cristatus* and short in *T*.* marmoratus*, Bouton, 1986; JWA, unpublished data); (2) the number of immature individuals in the water (frequent in *T. cristatus* and absent for *T. marmoratus,* Vallée, 1959; JWA, unpublished data); (3) the annual female fecundity (ca. 200 eggs in *T. cristatus* and ca. 400 eggs in *T. marmoratus*, Arntzen & Hedlund, 1990); (4) the skipping of annual breeding opportunities (infrequent in *T. cristatus* and regular in *T. marmoratus*, JWA, unpublished data), and (5) the preferential transmission of the maternally inherited “cristatus” mtDNA haplotypes (Arntzen et al., 2009). Field observations were made in spring when both species are in the water for breeding, and adult phenology will not have affected our results. Observations on juveniles were excluded. To test the hypothesis that factors under (3) and (4) are in equilibrium, we determined the dry weight of 50 *T. cristatus* and 39 *T. marmoratus* eggs, collected from ponds where hybrids or the counterpart species were known to be absent. The mean dry weight of *T. cristatus* eggs was 1.81 ± 0.34 mg (standard deviation), and for *T. marmoratus* eggs, it was 1.75 ± 0.33 mg, with no significant difference between the species (*t*‐test, *p* > .10). The energetic investments per egg appear similar for both species, and we assume that the high annual fecundity of *T. marmoratus* females (3) is offset by the skipping of annual breeding opportunities (4). While a significant reproductive asymmetry has been reported with hybrids predominantly transmitting the cristatus mtDNA haplotype (Arntzen et al., 2009), hybrids are relatively rare and the bias is minor. In all, numerical adjustments to the species counts were deemed unnecessary.

### 2.4 Species distribution models

The dependent variable in the study is species composition, in which *T. cristatus* and mixed populations with >50% *T. cristatus* are contrasted with pure and otherwise mixed *T. marmoratus* populations. The morphologically intermediate and easily recognizable *T. cristatus* × *T. marmoratus* F_1_ hybrids were taken to represent both species. Statistical evaluation was carried out with a logistic regression procedure in SPSS 20.0 (IBM Corp., 2011) in which (1) mixed ponds were downweighted relative to pure ponds on a 0.5 to unity scale, and (2) both species had equal impact in the analyses, in line with the number of populations investigated. One population with equal numbers of both species observed was not taken into consideration. For mixed populations, we also analyzed the arcsine‐transformed proportion of *T. marmoratus* against the environmental variables in linear regression.

Forty explanatory variables were taken from the following sources: BioClim variables from the WorldClim database (http://www.worldclim.org/bioclim); CORINE land cover by the European Environmental Agency (http://www.eea.europa.eu), in particular the classes arable land (code 12) and pasture (code 18); the soil texture variables AWC, BD, CFrag, Clay, Sand, Silt, and USDA from the Natural Resources Conservation Service database (http://www.nrcs.usda.gov/wps/portal/nrcs/site/national/ home); and AgLim1, Crusting, DR, EAWaC_top, ParMaDo1, Use (recoded as continuous, from cultivated to grassland to seminatural), UseDo, WR, and WRBLv1 from the European Soil Data Centre (http://esdac.jrc.ec.europa.eu). We considered IGN‐based maps on the distribution of hedgerows in 2006 and water bodies in 1993 and high‐resolution maps for altitude and forestation, provided by the “Conseil départemental de la Mayenne” (I. Brugioni, pers. comm.). The complementary forestation and hedgerow maps were combined. Variables were continuous with the exception of the categorical variables ParMaDo1, USDA, UseDo, and WRBLv1. Continuous variables were deselected from the analysis if not significant or if the coefficient of variation over the département Mayenne was <0.10. A single representative was chosen from clusters of variables with *r* ^2^ > .64 in a UPGMA analysis. The explanatory variables maintained are presented in Table 1. The conditions they describe were extracted over a circular area with a radius of 250 m around the pond, for all investigated *Triturus* populations. This scale is a compromise between mapping accuracy, average interpond distance in the study area, and the distance that adult amphibians reside, migrate, or disperse from ponds (Semlitsch & Bodie, 2003; Smith & Green, 2005). GIS analyses were carried out with ILWIS 3.3 (ILWIS, 2005).

**Table 1:** Environmental variables available for selection in logistic regression analysis

Explanatory variables	Description	Unit or classes (missing data)
AgLim	Dominant limitation to agricultural use	No limitation, gravelly, stony, lithic (3%)
Altitude	Elevation	Meter above sea level
AWC	Available water capacity	In seven classes
BD	Bulk density	In ten classes
Bio06	Minimum temperature of coldest month	Degrees Celsius
CFrag	Percentage of course fragments	In eight classes
Clay	Percentage clay	In eight classes
Corine 12	Arable field land cover	Percentage
Corine 18	Pasture land cover	Percentage
Crusting	Soil crusting class	Very weak, weak, moderate, strong, very strong
DR	Depth to roc	Shallow, moderate, deep, very deep
EAWaC_Top	Topsoil easily available water capacity	Low, medium, high, very high
Forestation	Presence/absence	Percentage land cover
Hedgerows	Presence/absence	Percentage land cover
ParMaDo1	Major group code for the dominant parent material	Categorical
Sand	Percentage sand	In eight classes
Silt	Percentage silt	In eight classes
USDA	Texture class	Silty clay, silt, loam, sandy loam
Use	Regrouped land use	Grassland, seminatural, cultivated
UseDo	Dominant land use	Categorical
Water bodies	Presence/absence	Percentage land cover
WR	Annual average soil water regime	Depth and period, from wet to dry in four classes
WrBLv1	Soil reference group	Categorical (5%)

For details on variables, see the Web sites listed in the text.

## 3 Results

**Figure 2:**
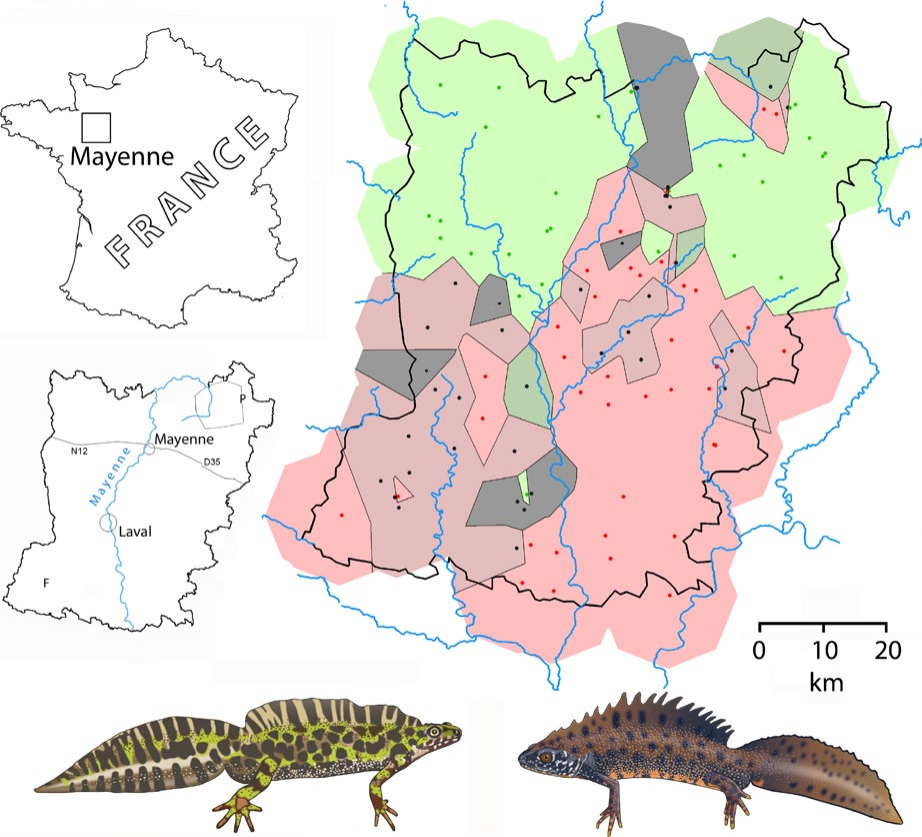
The distribution of *Triturus cristatus* (red dots, red shading, ventral side orange) and *Triturus marmoratus* (green dots, green shading, dorsal side green) in the département Mayenne in western France, presented in Dirichlet cells. The amount of species mixing is shown by light (<40%) and heavy shading (>40%) surrounding black dots. P—Pré‐en‐Pail, F—les Féages (see Figure 4). Drawn images are by Bas Blankevoort, copyright Naturalis Biodiversity Center

A total of *N *=* *183 adult *Triturus* newts were observed in 25 ponds. One hundred thirty‐five (74%) were *T. cristatus* in 20 ponds, 33 (18%) were *T. marmoratus* in nine ponds, and 15 (8%) were *T. cristatus* × *T. marmoratus* F_1_ hybrids in three ponds. A total of 1,155 eggs from 97 ponds were identified to the species. Approximately two‐third of the investigated eggs had the cristatus mtDNA haplotype, and one‐third had the marmoratus mtDNA haplotype. The number of ponds with the *Triturus* species composition determined was 102 with an average sample size of 13.1. Mixed populations are not significantly more or less frequent now (34%) than they were 35 years ago (28%) or 65 years ago (24%) (*G*‐test for independence, *p *>* *.10 in both cases). The survey results are plotted in Figure 2 as *T. cristatus* exclusively, 60% < *T. cristatus* < 100%, both species about equally frequent (i.e., in the 40%–60% range), 60% < *T. marmoratus* < 100%, and *T. marmoratus* exclusively. For ease of interpretation, the distribution results are presented in Dirichlet cells for which the spatial extrapolation does not exceed 10 km.

**Figure 3:**
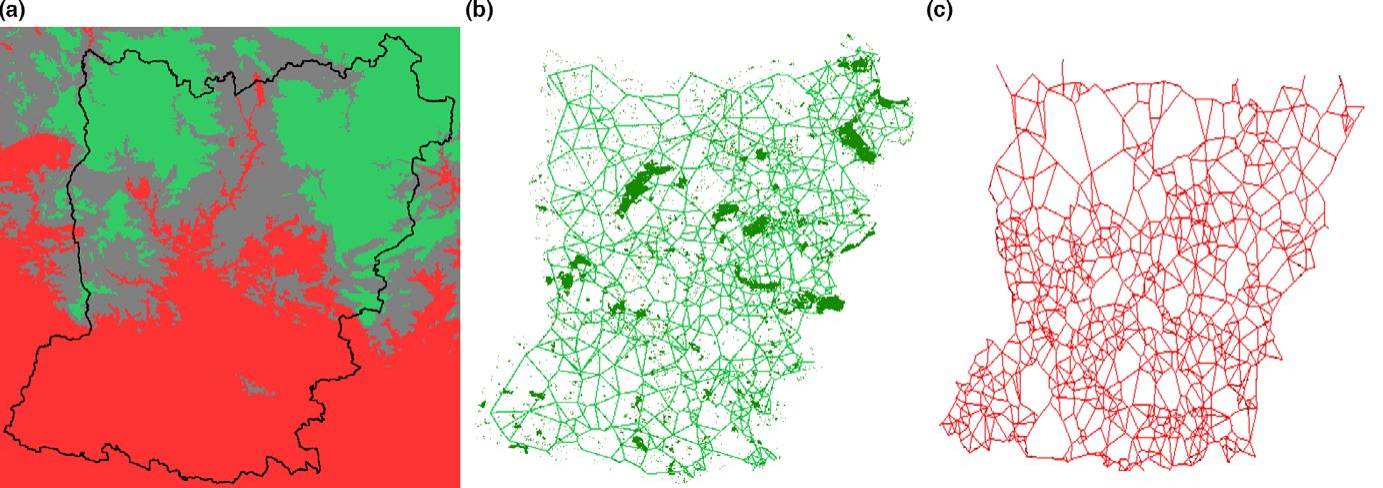
Environmental variables that significantly contribute to the model describing the distribution of *Triturus cristatus* vs. *Triturus marmoratus* in the département Mayenne in western France. Red and green colors are attributed to character states that support the presence of *T. cristatus* and *T. marmoratus*, respectively. (a) Altitude and the border of the département. The solid red color signifies the relatively flat area <113 m a.s.l. where the modeled probability of occurrence of *T. cristatus* is >60%; solid green signifies the more hilly area >161 m a.s.l. where the modeled probability of occurrence of *T. marmoratus* is >60%. (b) Forestation and Gabriel network of dense hedgerows. (c) Gabriel network of water bodies. The contributions to the logistic regression model (Table 2) are statistically significant as follows: altitude—*p *<* *.001, cover by forestation and hedgerows—*p *<* *.05, and water bodies—*p *<* *.01. For the linear regression model, the significances are altitude—*p *<* *.05, cover by forestation and hedgerows—*p *<* *.05, and water bodies—*p *<* *.10

Environmental variables that significantly contributed to the distribution model of *T. cristatus* vs. *T. marmoratus* were Altitude, Forest_hedgerows, and Water bodies, with an overall model fit of AUC = 0.847 ± 0.040 and kappa (κ) = 0.505 (Table 2). The three components to the model are illustrated in Figure 3. An alternative model, based just upon the variables Altitude and Forest_Hedgerows (cf. Arntzen & Wallis, 1991), yielded a fit of AUC = 0.778 ± 0.047 and κ = 0.373. For mixed populations, the proportion of *T. marmoratus* was correlated with the same parameters (Table 2).

**Table 2:** Models for the distribution of *Triturus cristatus*‐ vs. *Triturus marmoratus*‐dominated populations in the département Mayenne, France

Selected variables	(A) Logistic regression model		(B) Linear regression model
	Arntzen and Wallis (1991)	Present study	
Altitude	−0.019	−0.017	0.117
Forest_hedgerows	−0.033	−0.025	0.248
Water bodies	2.328	NA	−11.521
Constant	2.764	3.030	−75.104
Number of populations	101	154	35
Model fit			
AUC ± SD	0.847 ± 0.040	0.778 ± 0.047	NA
Kappa	0.51	0.37	NA
Pearson *r*	NA	NA	0.50

Explanatory variables available for selection are listed in Table 1. (A) Logistic regression analysis on a binary classification of species composition. (B) Linear regression analysis on arcsine‐transformed proportion of *T. marmoratus* to the total population.

NA—not available for selection or not applicable.

Throughout the département Mayenne, 145 recorded *Triturus* ponds were revisited with a 35‐year interval. Most ponds had either lost their depth or disappeared completely (*n *=* *103, 71%). Forty‐two ponds persisted, of which 20 with *Triturus* newts (14%) and 22 without *Triturus* newts (15%). In the section of Mayenne north of the N12 and D35 roads, 64 amphibian ponds were revisited with the same time interval, and only 11 of these ponds persisted (17%). In the Pré‐en‐Pail area, 99 amphibian ponds were revisited with an 18‐year interval. Forty‐three ponds persisted (43%). The occupancy by *Triturus* newts in this area was 46% in 1997 and 16% in 2015.

## 4 Discussion

The notion that hybrid zones may move over the landscape is relatively new to science (for a review see Buggs, 2007). The argument for movement is mostly inferential (Buggs, 2007; see also Carling & Zuckerberg, 2011; Charpentier et al., 2012; Gay, Crochet, Bell, & Lenormand, 2008; Krosby & Rohwer, 2009; Leafloor, Moore, & Scribner, 2013). Examples of direct observation on hybrid zone dynamics are rare, because of the time frame involved (Buggs, 2007; see also Engebretsen, Barrow, Rittmeyer, Brown, & Moriarty Lemmon, 2016; Ouanes, Bahri‐Sfar, Ben Hassine, & Bonhomme, 2011; Roy, O'Connor, & Green, 2012; Smith et al., 2013; Taylor, Curry, White, Ferretti, & Lovette, 2014). We here document by direct observation a unique case of a hybrid zone that first moved and then stabilized in historical times.

*Triturus cristatus* and *T. marmoratus* are hybridizing species in which the frequency of hybrids in the adult F_1_ class averages at 4% (Arntzen et al., 2009; Vallée, 1959). The fertility of the F_1_ hybrids is low, and interspecific gene flow is limited. The *T. cristatus* and *T. marmoratus* contact classifies as a traditional “mosaic hybrid zone” (*sensu* Harrison & Rand, 1989) on account of the following: (1) the bimodal (or “trimodal,” Gay et al., 2008) genetic profile displayed, (2) the pronounced ecological differentiation expressed by the species, and (3) the patchy distribution pattern at which they engage. Following the initial survey on *Triturus* newts in Mayenne shortly after the Second World War (Vallée, 1959), *T. cristatus* has superseded *T. marmoratus* over a large portion of the département (Arntzen & Wallis, 1991; Schoorl & Zuiderwijk, 1981) (Figures 1 and 2). Hybrid zone movement and species displacement are thought to be triggered or accelerated by anthropogenic change, namely the removal of hedgerows in the postwar period that allowed the fairly aquatic *T. cristatus* to supersede *T. marmoratus*, which is a more terrestrial species with preferences for hilly and forested terrain (Schoorl & Zuiderwijk, 1981).

*Triturus cristatus* has a northerly European distribution, and *T. marmoratus* has a distribution in southern France and Iberia. In spite of this general pattern, the area *T. cristatus* took over is in the *south* of Mayenne, whereas the distribution of *T. marmoratus* over the *northern* part of Mayenne remained largely unchanged (Figures 1 and 2). The effective dispersal of *T. cristatus* was ca. 1 km per year (Arntzen & Wallis, 1991). *Triturus marmoratus* was not wiped out completely from the south of the département but persisted in local populations at low frequency, often in syntopy with *T. cristatus* and with ongoing hybridization and introgression. The continued syntopy of the species is not surprising given the newts' longevity, with an observed maximum of 14 years in *T. cristatus* and *T. marmoratus* and 17+ years in F_1_ hybrids (Francillon‐Vieillot, Arntzen, & Géraudie, 1990). A wider analysis of the distribution of *Triturus* species and the landscape suggests that the dispersal route taken by *T. cristatus* is along the Loire river (JWA, unpublished) and that *T. cristatus* entered Mayenne from the east. An eastern point of entrance is supported by Vallée's (1959) distribution data (Figure 1a). From here, the species dispersed westwards and northwards, both prior to 1950. The northernmost *T. cristatus* locality near Pré‐en‐Pail was initially interpreted as an introduction (cf. footnote to Table 2 in Vallée, 1959), but molecular genetic data indicate that the local presence is natural. The arguments underlying this claim are as follows: (1) assignment tests indicate a closer genetic relationship with other northern *T. cristatus* populations than with the presumed source of the introduction near the city of Laval in the center of the département, and (2) the genetic variability of the Pré‐en‐Pail *T. cristatus* population is such that the propagule size of an introduction must have been large, which is unlikely (Arntzen et al., 2010). The alternative scenario is the northward dispersal of *T. cristatus* to have reached the Pré‐en‐Pail area along the Mayenne river. The existence (or past existence) of this dispersal corridor is supported by observations to the northwest (first survey, Figure 1a) and northeast of Mayenne city (third survey, Figure 2). We assume that at the second survey, this connection remained unnoticed due to sparse sampling (Figure 1b).

The present‐day distribution of species and hybrids is comparable to that of the second survey (Figure 2). Southern localities with just *T. marmoratus* (“marmoratus enclaves”) are rare. The local absence of *T. cristatus* may reflect small sample sizes and stochastic effects in small populations. The most likely places for genuine marmoratus enclaves are the large forests in the south of Mayenne. Unfortunately, access to forests was frequently denied, and forest ponds are underrepresented in our survey. Mixed *T. cristatus–T. marmoratus* populations are frequent, especially along the Mayenne river and in the southwest of the département. Given the arrival of *T. cristatus* in the east of the département and its widespread advance, it is tempting to interpret the current pattern (with more mixed populations in the southwest than in the southeast of the département) as reflecting the colonization and superseding process (Figure 2). It is puzzling though why the same pattern was not found already at the second survey (Figure 1b). It is equally tempting to attribute the northward shift of the species border to climate warming, but this counteracts the fact that it is *T. marmoratus* from southern France and Iberia, presumably well adapted to high temperatures, that is the receding party (cf. Taylor, Larson, & Harrison, 2015; Taylor et al., 2014) and other parameters appear to be involved. A remarkable feature is the natural occurrence of a syntopic/allotopic *T. cristatus* population in the northeast of the département. This occurrence was observed at all three surveys and goes to show that an isolated occurrence or enclave may actively be formed as an expansion of the range ahead of the main distribution.

**Figure 4:**
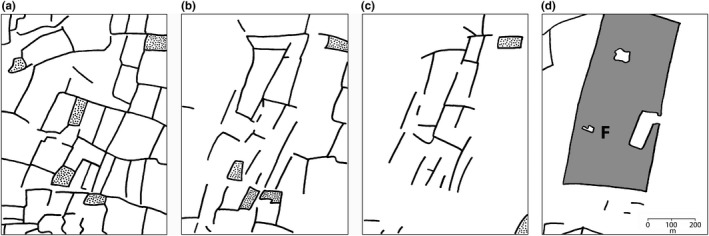
Example of the postwar disappearance of the “bocage” aspect from the landscape in western France. Lines are hedgerows, stippled areas are orchards, and shading is forestation. (a) 1949, (b) 1977, (c) 1992 (from Arntzen, 1998, with permission), and (d) situation in 2013 (from Geoportail at http://tab.geoportail.fr). The local situation in the southwest of the département Mayenne around farmhouse ‘les Féages’ (*F*) is peculiar as hedgerows initially remained (a–c), but were not maintained (d)

The study of a wide variety of environmental variables yielded no new insights into the ecological parameters that determine the mutual *T. cristatus*/*T. marmoratus* distribution in Mayenne, except for the density of water bodies that was identified as a significant contributor to the best distribution model, in addition to altitude and the density of hedgerows and forestation (cf. Arntzen & Wallis, 1991). Of the parameters that contributed to the species model, the density of hedgerows is most likely to change over time. Indeed, the typical “bocage” (dense network of mature hedgerows) landscape has largely been eradicated to allow for larger field sizes, in particular in the flat, southern part of Mayenne. For a dramatic illustration of the magnitude of the change, see Figure 4. The flat southern part of Mayenne with a high density of water bodies appears particularly suited for (the advance of) *T. cristatus*, the more aquatic of the two species. Its relative advantage over *T. marmoratus*, the more terrestrial species, was boosted by the removal of hedgerows. Other habitat features are likely to play a role, such as the preference of *T. marmoratus* for springs and well‐vegetated ponds (Schoorl & Zuiderwijk, 1981), but parameters like these are difficult to model in a spatial context. In mixed ponds, the contribution of *T. cristatus* to the total population was negatively correlated with the density of hedgerows around the pond. Accordingly, the cutting of hedgerows may well have been the prime factor that caused the hybrid zone to move.

As noted, the changes we documented in the distribution of *T. cristatus* vs. *T. marmoratus* over Mayenne from the second to the third survey are relatively small. *Triturus cristatus* has not markedly continued its advance over *T. marmoratus,* and mixed populations are equally frequent now as they were 35 and 65 years ago. It is therefore fair to judge that the *T. cristatus* vs. *T. marmoratus* moving hybrid zone has come to a standstill. Prime candidates for forming a barrier to the further advance of *T. cristatus* are the ecological variables that help to explain the current species distribution, namely altitude (*p *<* *.001), pond density (*p *<* *.01), and hedgerow and forestation (*p *<* *.05) (Figure 3). The transition from flat to hilly terrain at 113–161 m a.s.l. that sets the species apart also separates the south of the département from the north (Figure 3a). This parameter to some extent represents the other selected parameters because the amount of shelter provided by hedgerows and other small landscape elements (favoring *T. marmoratus*) is higher in hilly terrain than in flat areas and more ponds (favoring *T. cristatus*) are found in flat than in hilly areas. To disentangle the relative contribution of altitude, forestation and pond density will require data at either a larger scale (e.g., countrywide), or at a smaller scale where species interactions are most intense. An alternative explanation for hybrid zone fixation is the pond loss that followed agricultural intensification and the change from pasture to arable land use. In Mayenne, the loss of *Triturus* ponds was 82% in the 1950–1979 interval (Schoorl & Zuiderwijk, 1981) and another 86% in the 1979–2015 interval. This amounts to a steady loss of 5.5% of ponds per year. This rate is substantially higher than the annual loss averaging at 0.8% in United Kingdom (Wood, Greenwood, & Agnew, 2003) and 3.5% in northwestern France (Curado, Hartel, & Arntzen, 2011). The decline will have compromised the dense network of ponds that is required for a healthy *Triturus* population network (Halley, Oldham, & Arntzen, 1996). Without ponds as “stepping stones,” new areas cannot be colonized.

## Conflict of Interest

None declared.

## References

[ece32676-bib-0001] Abbott, R. , Albach, D. , Ansell, S. , Arntzen, J. W. , Baird, S. J. , Bierne, N. , … Zinner, D. (2013). Hybridization and speciation. Journal of Evolutionary Biology, 26, 229–246. 23323997 10.1111/j.1420-9101.2012.02599.x

[ece32676-bib-0002] Arntzen, J. W. (1978). Some hypotheses on postglacial migrations of the Fire‐bellied toad, *Bombina bombina* (Linnaeus) and the Yellow‐bellied toad, *Bombina variegata* (Linnaeus). Journal of Biogeography, 5, 339–345.

[ece32676-bib-0003] Arntzen, J. W. (1996). Parameters of ecology and scale integrate the gradient and mosaic models of hybrid zone structure in *Bombina* toads and *Triturus* newts. Israel Journal of Zoology, 42, 111–119.

[ece32676-bib-0004] Arntzen, J. W. (1998). Les tritons en Mayenne. Biotopes, 53(16), 61–67.

[ece32676-bib-0005] Arntzen, J. W. , Burke, T. , & Jehle, R. (2010). Estimating the propagule size of a cryptogenic crested newt population. Animal Conservation, 13(s1), 74–81.

[ece32676-bib-0006] Arntzen, J. W. , & Espregueira Themudo, G. (2008). Environmental parameters that determine species geographical range limits as a matter of time and space. Journal of Biogeography, 35, 1177–1186.

[ece32676-bib-0007] Arntzen, J. W. , & Hedlund, L. (1990). Fecundity of the newts *Triturus cristatus*,* T. marmoratus* and their natural hybrids in relation to species coexistence. Ecography, 13, 325–332.

[ece32676-bib-0008] Arntzen, J. W. , Jehle, R. , Bardakci, F. , Burke, T. , & Wallis, G. P. (2009). Asymmetric viability of reciprocal‐cross hybrids between crested and marbled newts (*Triturus cristatus* and *T. marmoratus*). Evolution, 63, 1191–1202. 19154385 10.1111/j.1558-5646.2009.00611.x

[ece32676-bib-0009] Arntzen, J. W. , & Wallis, G. P. (1991). Restricted gene flow in a moving hybrid zone of the newts *Triturus cristatus* and *T. marmoratus* in western France. Evolution, 45, 805–826. 10.1111/j.1558-5646.1991.tb04352.x 28564049

[ece32676-bib-0010] Bouton, N. (1986). Données sur la migration de *Triturus cristatus* et *T. marmoratus* (Urodela, Salamandridae) dans le département de la Mayenne (France). Bulletin Société Herpetologique de France, 40, 43–51.

[ece32676-bib-0011] Buggs, R. J. A. (2007). Empirical study of hybrid zone movement. Heredity, 99, 301–312. 17611495 10.1038/sj.hdy.6800997

[ece32676-bib-0012] Carling, M. D. , & Zuckerberg, B. (2011). Spatio‐temporal changes in the genetic structure of the Passerina bunting hybrid zone. Molecular Ecology, 20, 1166–1175. 21232074 10.1111/j.1365-294X.2010.04987.x

[ece32676-bib-0013] Charpentier, M. J. E. , Fontaine, M. C. , Cherel, E. , Renoult, J. P. , Jenkins, T. , Benoit, L. , … Tung, J. (2012). Genetic structure in a dynamic baboon hybrid zone corroborates behavioural observations in a hybrid population. Molecular Ecology, 21, 715–731. 21988698 10.1111/j.1365-294X.2011.05302.x

[ece32676-bib-0014] Curado, N. , Hartel, T. , & Arntzen, J. W. (2011). Amphibian pond loss as a function of landscape change—A case study over three decades in an agricultural area of northern France. Biological Conservation, 144, 1610–1618.

[ece32676-bib-0015] Dejean, T. , Miaud, C. , & Schmeller, D. (2010). Protocole d'hygiène pour limiter la dissémination de la Chytridiomycose lors d'interventions sur le terrain. Bulletin de la Société Herpétologique de France, 134, 47–50.

[ece32676-bib-0016] Engebretsen, K. N. , Barrow, L. N. , Rittmeyer, E. N. , Brown, J. M. , & Moriarty Lemmon, E. (2016). Quantifying the spatiotemporal dynamics in a chorus frog (*Pseudacris*) hybrid zone over 30 years. Ecology and Evolution, 6, 5013–5031. 27547330 10.1002/ece3.2232 PMC4979724

[ece32676-bib-0017] Francillon‐Vieillot, H. , Arntzen, J. W. , & Géraudie, J. (1990). Age, growth and longevity of sympatric *Triturus cristatus*,* T. marmoratus* and their hybrids (Amphibia, Urodela): a skeletochronological comparison. Journal of Herpetology, 24, 13–22.

[ece32676-bib-0018] Gay, L. , Crochet, P. A. , Bell, D. A. , & Lenormand, T. (2008). Comparing clines on molecular and phenotypic traits in hybrid zones: A window on tension zone models. Evolution, 62, 2789–2806. 18752618 10.1111/j.1558-5646.2008.00491.x

[ece32676-bib-0019] Halley, J. M. , Oldham, R. S. , & Arntzen, J. W. (1996). Predicting the persistence of amphibian populations with the help of a spatial model. Journal of Applied Ecology, 33, 455–470.

[ece32676-bib-0020] Harrison, R. G. (1990). Hybrid zones: windows on evolutionary process. Oxford Surveys in Evolutionary Biology, 7, 69–128.

[ece32676-bib-0021] Harrison, R. G. , & Rand, D. M. (1989). Mosaic hybrid zone and the nature of species boundaries In Otte D., & Endler J. A. (Eds.), Speciation and its Consequences (pp. 111–133). Sunderland, MA: Sinauer Associates Inc. Publishers.

[ece32676-bib-0022] Hewitt, G. M. (1988). Hybrid zones—natural laboratories for evolutionary studies. Trends in Ecology & Evolution, 3, 158–167. 21227192 10.1016/0169-5347(88)90033-X

[ece32676-bib-0023] Hewitt, G. M. (1999). Post‐glacial re‐colonization of European biota. Biological Journal of the Linnean Society, 68, 87–112.

[ece32676-bib-0024] IBM Corp (2011). IBM SPSS statistics for windows, version 20.0. Armonk, NY: IBM Corp.

[ece32676-bib-0025] ILWIS (2005). ILWIS—Integrated land and water information system. Remote sensing and GIS software. Enschede, The Netherlands: ITC.

[ece32676-bib-0026] Johnson, M. L. , Berger, L. , Philips, L. , & Speare, R. (2003). Fungicidal effects of chemical disinfectants, UV light, desiccation and heat on the amphibian chytrid *Batrachochytrium dendrobatidis* . Diseases of Aquatic Organisms, 57, 255–260. 14960039 10.3354/dao057255

[ece32676-bib-0027] Krosby, M. , & Rohwer, S. (2009). A 2000 km genetic wake yields evidence for northern glacial refugia and hybrid zone movement in a pair of songbirds. Proceedings of the Royal Society of London B: Biological Sciences, 276, 615–621. 10.1098/rspb.2008.1310 PMC2660942 18986973

[ece32676-bib-0028] Leafloor, J. O. , Moore, J. A. , & Scribner, K. T. (2013). A hybrid zone between Canada Geese (*Branta canadensis*) and Cackling Geese (*B. hutchinsii*). The Auk, 130, 487–500.

[ece32676-bib-0029] Littlejohn, M. J. , & Roberts, J. D. (1975). Acoustic analysis of an intergrade zone between two call races of the *Limnodynastes tasmaniensis* complex (Anura: Leptodactylidae) in south‐eastern Australia. Australian Journal of Zoology, 23, 113–122.

[ece32676-bib-0030] Mallet, J. (2005). Hybridization as an invasion of the genome. Trends in Ecology and Evolution, 20, 229–237. 16701374 10.1016/j.tree.2005.02.010

[ece32676-bib-0031] Ouanes, K. , Bahri‐Sfar, O. L. , Ben Hassine, O. K. , & Bonhomme, F. (2011). Expanding hybrid zone between *Solea aegyptiaca* and *Solea senegalensis*: Genetic evidence over two decades. Molecular Ecology, 20, 1717–1728. 21426433 10.1111/j.1365-294X.2011.05034.x

[ece32676-bib-0032] Roy, J. S. , O'Connor, D. , & Green, D. M. (2012). Oscillation of an anuran hybrid zone: Morphological evidence spanning 50 years. PLoS One, 26, e52819. 10.1371/journal.pone.0052819 PMC3530495 23300785

[ece32676-bib-0033] Schmeller, D. S. , Loyau, A. , Dejean, T. , & Miaud, C. (2011). Using amphibians in laboratory studies: Precautions against the emerging infectious disease chytridiomycosis. Laboratory Animals, 45, 25–30. 21075827 10.1258/la.2010.010101

[ece32676-bib-0034] Schoorl, J. , & Zuiderwijk, A. (1981). Ecological isolation in *Triturus cristatus* and *Triturus marmoratus* (Amphibia: Salamandridae). Amphibia‐Reptilia, 1, 235–252.

[ece32676-bib-0035] Semlitsch, R. D. , & Bodie, J. R. (2003). Biological criteria for buffer zones around wetlands and riparian habitats. Conservation Biology, 17, 1219–1228.

[ece32676-bib-0036] Smith, M. A. , & Green, D. M. (2005). Dispersal and the metapopulation paradigm in amphibian ecology and conservation: Are all amphibian populations metapopulations? Ecography, 28, 110–128.

[ece32676-bib-0037] Smith, K. L. , Hale, J. M. , Gay, L. , Kearney, M. , Austin, J. J. , Parris, K. M. , & Melville, J. (2013). Spatio‐temporal changes in the structure of an Australian frog hybrid zone: A 40‐year perspective. Evolution, 67, 3442–3454. 24299399 10.1111/evo.12140

[ece32676-bib-0038] Szymura, J. M. (1993). Analysis of hybrid zones with *Bombina* In Harrisson R. G. (Ed.), Hybrid zones and the evolutionary process (pp. 261–289). New York, NY: Oxford University Press.

[ece32676-bib-0039] Taberlet, P. , Fumagalli, L. , Wust‐Saucy, A. G. , & Cosson, J. F. (1998). Comparative phylogeography and postglacial colonization routes in Europe. Molecular Ecology, 7, 453–464. 9628000 10.1046/j.1365-294x.1998.00289.x

[ece32676-bib-0040] Taylor, S. A. , Curry, R. L. , White, T. A. , Ferretti, V. , & Lovette, I. (2014). Spatiotemporally consistent genomic signatures of reproductive isolation in a moving hybrid zone. Evolution, 68, 3066–3081. 25138643 10.1111/evo.12510

[ece32676-bib-0041] Taylor, S. A. , Larson, E. L. , & Harrison, R. G. (2015). Hybrid zones: Windows on climate change. Trends in Ecology and Evolution, 30, 398–406. 25982153 10.1016/j.tree.2015.04.010 PMC4794265

[ece32676-bib-0042] Taylor, S. A. , White, T. A. , Hochachka, W. M. , Ferretti, V. , Curry, R. L. , & Lovette, I. (2014). Climate‐mediated movement of an avian hybrid zone. Current Biology, 17, 671–676. 10.1016/j.cub.2014.01.069 24613306

[ece32676-bib-0043] Vallée, L. (1959). Recherches sur *Triturus blasii* de l'Isle, hybride naturel de *Triturus cristatus* Laur. × *Triturus marmoratus* Latr. Mémoires de la Société Zoologique de France, 31, 1–95.

[ece32676-bib-0044] Vörös, J. , Mikulíček, P. , Major, A. , Recuero, E. , & Arntzen, J. W. (2016). Phylogeographic analysis reveals northerly refugia for the riverine amphibian *Triturus dobrogicus* (Caudata: Salamandridae). Biological Journal of the Linnean Society, 119, 974–991. doi: 10.1111/bij.12866

[ece32676-bib-0045] Walsh, P. S. , Metzger, D. A. , & Higuchi, R. (1991). Chelex 100 as a medium for simple extraction of DNA for PCR‐based typing from forensic material. BioTechniques, 10, 506–513. 1867860

[ece32676-bib-0046] Wielstra, B. , & Arntzen, J. W. (2012). Postglacial species displacement in *Triturus* newts deduced from asymmetrically introgressed mitochondrial DNA and ecological niche models. BMC Evolutionary Biology, 12, 161. doi: 10.1186/1471‐2148‐12‐161 22935041 10.1186/1471-2148-12-161 PMC3520116

[ece32676-bib-0047] Wielstra, B. , Crnobrnja‐Isailović, J. , Litvinchuk, S. N. , Reijnen, B. T. , Skidmore, A. K. , Sotiropoulos, K. , … Arntzen, J. W. (2013). Tracing glacial refugia of *Triturus* newts based on mitochondrial DNA phylogeography and species distribution modeling. Frontiers in Zoology, 10, 13. 23514662 10.1186/1742-9994-10-13 PMC3608019

[ece32676-bib-0048] Wood, P. J. , Greenwood, M. T. , & Agnew, M. D. (2003). Pond biodiversity and habitat loss in the UK. Area, 35, 206–216.

